# Quality by design approach of apocynin loaded clove oil based nanostructured lipid carrier as a prophylactic regimen in hemorrhagic cystitis in vitro and in vivo comprehensive study

**DOI:** 10.1038/s41598-024-68721-z

**Published:** 2024-08-19

**Authors:** Amir Elsayed Maghrabia, Mariza Fouad Boughdady, Sherry Mohamed Khater, Irhan Ibrahim ِِAbu Hashim, Mahasen Mohammed Meshali

**Affiliations:** 1 Department of Pharmaceutics, Faculty of Pharmacy, Mansoura University, Mansoura, 35516 Egypt; 2 Department of Pharmacy, Urology and Nephrology Center, Mansoura University, Mansoura, 35516 Egypt; 3 Department of Clinical Pathology, Urology and Nephrology Center, Mansoura University, Mansoura, 35516 Egypt

**Keywords:** Apocynin, Clove oil, Nanostructured lipid carrier, Bladder cancer, Cystitis, Phytopharmaceuticals, Pharmaceutics, Nanomedicine

## Abstract

Apocynin (APO) is a naturally occurring acetophenone with eminent anti-inflammatory and anti-oxidant peculiarities. It suffers from poor bioavailability due to low aqueous solubility. Herein, APO was loaded in a Clove oil (CO) based Nanostructured lipid carrier (NSLC) system using a simple method (ultrasonic emulsification) guided by a quality-by-design approach (2^3^ full factorial design) to optimize the formulated NSLCs. The prepared NSLCs were evaluated regarding particle size (PS), polydispersity index (PDI), zeta potential (ZP), and entrapment efficiency (EE%). The optimal formula (F2) was extensively investigated through transmission electron microscope (TEM), Fourier transform infrared (FT-IR) spectroscopy, Differential scanning calorimetry (DSC), X-ray diffractometry (XRD), in vitro release, and stability studies. Cytotoxicity against human urinary bladder carcinoma (T24) cell line and in vivo activity studies in rats with induced cystitis were also assessed. The results disclosed that the optimal formula (F2) had PS of 214.8 ± 5.8 nm with EE% of 79.3 ± 0.9%. F2 also exhibited a strong cytotoxic effect toward the T24 cancer cells expressed by IC50 value of 5.8 ± 1.3 µg/mL. Pretreatment with the optimal formula (orally) hinted uroprotective effect against cyclophosphamide (CP)-induced hemorrhagic cystitis (HC) in rat models, emphasized by histopathological, immunohistochemical, and biochemical investigations. In consideration of the simple fabrication process, APO-loaded CO-based NSLCs can hold prospective potential in the prophylaxis of oncologic and urologic diseases.

## Introduction

Phytopharmaceuticals are small molecular compounds found in edible plants that exhibit unique characteristics making them therapeutically active^1^. Some of them play a vital role in scavenging free radicals, regulating enzyme activity, and chelating metals^2^. In this study, for the first time, two phytopharmaceuticals, Apocynin (APO) and Clove oil (CO), with their dual antioxidant and anti-inflammatory properties, were combined in one formula^3^.

APO is a methoxy-substituted catechol; retrieved from the roots of *Apocynum cannabinum and Picrorhiza kurroa*^4^. *Apocynum cannabinum*, commonly known as Canadian hemp. It is native to the United States and southern Canada, but most abundant in the upper Mississippi River Valley and east to the Atlantic coast. It has long been used by certain Indian tribes in the treatment of dropsies and as an antidote to snakebites^5^. *Picrorhiza kurroa*, is a small perennial herb in the family *scrophulariaceae*. It is a therapeutic herb found in the alpine Himalayan region. It has various pharmacological characteristics such as hepatoprotective, antiasthmatic, antiallergic, antioxidant, and anticancer potential^6,7^. Apo has been demonstrated to be an effective inhibitor of NADPH oxidase along with its function to reduce the production of reactive oxygen species (ROS) in numerous cells. Moreover, it also has a repressive impact on apoptotic pathways and pro-inflammatory cytokines^8^. Therefore, APO has shown therapeutic applications in many inflammatory diseases^9^. However, its clinical impact is restricted due to poor oral bioavailability (10%)^10^. Various nanocarriers have been developed to increase the oral bioavailability of APO by utilizing different polymers; such as polylactic co-glycolic acid (PLGA) and chitosan (CH)^11–14^.

CO is a natural oil extracted from the spice *Syzigum aromaticum*^15^. It is recognized with some therapeutic properties, such as antioxidant, anti-inflammatory, and anti-aging, as well as its activity against pathogenic organisms. These effects are attributed to the presence of unsaturated phenolic compounds; mainly eugenol^16^. Eugenol is volatile and chemically unstable in the presence of air and light, so its encapsulation could be a perfect choice to safeguard the bioactive substance^2^.

Lipids are frequently used for oral delivery of lipophilic active ingredients, as they promote drug absorption in the gastrointestinal tract (GIT). They also increase mucosal adhesion and henceforth GIT residence. In addition, lipid NPs shield the loaded pharmaceuticals against chemical and enzymatic deterioration^17^. In this regard, lipid-based nanocarriers are a viable formulation strategy for APO and CO. They can overcome all of their unfavorable characteristics by shielding the bioactive substance and enhancing cellular bioavailability^18,19^. Various nano lipid-based carriers have been formulated and studied. Among them, nanostructured lipid carriers (NSLCs) have been recognized as superior to others in oral delivery^20^. NSLCs have been one of the promising carriers for broad applications for oral bioavailability enhancement^21,22^. They have been successfully applied to improve the bioavailability of bio-actives^23^, and target sites such as the brain^24^, liver^25^, and cancer cells^26^. NSLCs involve a lipid core comprising mixed solid and liquid lipids dispersed in an aqueous emulsifier solution with a nanoscale size range^27^**.** The presence of solid and liquid lipids in their structure empowers greater entrapment efficiency (EE%) and long-term colloidal stability unlike other solid lipid-based formulations^28^. Gelucires are inert semisolid waxy amphiphilic excipients with surface active properties that spontaneously form a fine dispersion or emulsion upon exposure to water. They are considered an alternative to other polymers employed in sustained-release formulations because of their potential biocompatibility and biodegradability^29^. In this study, Gelucire 43/01 was selected as a solid lipid due to its extreme hydrophobicity (HLB = 1), low density, and melting point above the body temperature^30^. CO served as a liquid lipid for the preparation of the NSLCs. To coat these NSLCs, a shell of chitosan (CH) was applied as an outer mucoadhesive coat^31^. With the help of a simple and reproducible technique based on pre-emulsion formation (ultrasonication), APO-loaded CO-based NSLCs were formulated^32^**.**

To judge the biological effectiveness of the formulated NSLCs wisely, the in vitro cytotoxicity on the bladder cancer (T24) cell line and the in vivo activity on an inflammatory module were assessed. Hemorrhagic cystitis (HC) is a serious inflammatory injury that affects the urinary bladder. It is characterized by bleeding from the bladder mucosa. Numerous causes for this condition have been identified however, exposure to cyclophosphamide (CP) remains the most imperative of all^33^. CP is a chemotherapeutic agent that is constantly used for pelvic malignancies. Intraperitoneal injection of CP in rats has been proposed to develop HC manifested as visceral discomfort, oxidative stress, and acute inflammation. The urotoxicity of CP roots from renal excretion of the corrosive hepatic metabolite (acrolein). The overabundance of acrolein within the bladder induces oxidative stress reactions, ensuing urothelium ulceration^34,35^. A variety of treatment modalities have been established for managing CP-induced HC, but none is consistently effective, except for using Mercaptoethane sulfonate (Mesna) as a prophylaxis^36,37^. Ihsan and his co-authors reported that antioxidants could alleviate oxidative stress by diminishing ROS levels in bladder tissues^38^. As far as our knowledge, APO’s impact on CP-induced HC has not been studied before. From this perspective, the authors assess the potential of using the novel fabricated APO-loaded CO-based NSLCS as a prophylactic treatment against bladder oxidative injury.

In light of the aforementioned information, the present study was carried out to build a CO-based NSLC system loaded with APO to improve the oral delivery of these miraculous phytopharmaceuticals using a simple method (ultrasonic emulsification). To fulfill this goal, design of experiments (DOE) approaches have been implemented currently, where the “best solution” for an ideal formulation can be estimated by conducting a few trials^39^. Ultimately, the uroprotective impact of the optimized NSLCs loaded with the two golden phytopharmaceuticals (APO and CO) was assessed against CP-induced HC in rats.

## Materials and methods

### Materials

APO was purchased from Sigma-Aldrich (Saint Louis, MO, USA). CO was obtained from FUJIFILM WAKO Pure Chemical Corporation. Both Tween 80 (CAS NO. 9005-65-6) and CH (CAS NO. 9012-76-4), low molecular weight (50,000–190,000 Da) and high degree of deacetylation (75–85%)) were purchased from Sigma-Aldrich (Saint-Louis, MO, USA). Gattefosse (St Priest, Cedex, France) graciously sent GE 43/01 (CAS NO. 85665-33-4, HLB: 1, Melting point: 43 °C) as a gift. El-Nasr Pharmaceutical Chemical Co. (Cairo, Egypt) provided an analytical grade of glacial acetic acid, hydrochloric acid 33% (HCl), disodium hydrogen phosphate (Na_2_HPO_4_), and monobasic potassium phosphate (KH_2_PO_4_). Cyclophosphamide (CP) (Endoxan® 200 mg) and Mesna (Uromitexan® 400 mg/4 mL) were purchased from Baxter Oncology Healthcare Pty Limited, USA. Rat ELISA kits of glutathione (GSH), superoxide dismutase (SOD), total nitric oxide (NO), and malondialdehyde (MDA) were procured from Cusabio, USA. In addition, anti-nuclear factor-κB (NF-κB/p65/catalog number 14-6731, eBioscience, Germany) and anti-cyclooxygenase-2 (COX-2/rabbit pAb, service bio, Belgium) were procured also.

### Methods

#### Design of experiment (DOE)

The relationship between critical process parameters (CPPs) and critical quality attributes (CQAs) was properly defined using a 2^3^ full factorial design. APO amount (XA), GE amount (XB), and CH concentration (XC) were the three CPPs. They were investigated at two levels, which are represented by the coded values of − 1 (low levels) and + 1 (high levels) in Table 1. Important CQAs like maximal drug entrapment efficiency (EE%), zeta potential (ZP) and minimal particle size (PS), polydispersity index (PDI), were kept in mind for dosage optimization and enhancement of both stability and bioavailability. Eight formulae, each with three runs, were created by the design and were then characterized for four CQAs: EE%, PS, PDI, and ZP.

**Table 1:** 2^3^ full factorial design and CPPs levels.

CPPs		Units	Minimum	Maximum	Coded low	Coded high
X_A_	APO amount	mg	10	20	− 1	+ 1
X_B_	GE amount	mg	50	100	− 1	+ 1
X_C_	CH concentration	% (w/v)	0.5	1	− 1	+ 1

1 mL of clove oil (CO) and 1% of Tween 80 were constant in all formulae.

*CPPs* critical process parameters, *APO* apocynin, *GE* gelucire 43/01, *CH* chitosan.

The first order polynomial regression equation:

$${\text{Y}} = \, \beta_{0} + \, \beta_{{1}} {\text{XA}} + \, \beta_{{2}} {\text{XB}} + \, \beta_{{3}} {\text{XC}} + \, \beta_{{4}} {\text{XAXB}} + \, \beta_{{5}} {\text{XAXC}} + \, \beta_{{6}} {\text{XBXC}} + \, \beta_{{7}} {\text{XAXBXC}}.$$

Y was the dependent variable (CQA) in this scenario. The independent variables (CPPs) are XA, XB, and XC. The arithmetic mean response of the eight runs is represented as β_0_. The coefficients of the linear equation are β_1_, β_2_, and β_3_. The coefficients of interaction between the two CPPs are β_4_, β_5_, and β_6_, respectively. β_7_ represents the interaction coefficients between the three CPPs.

### Preparation of APO-loaded CO-based NSLCs

NSLCs were prepared by ultrasonic emulsification technique^40^. The first step for NSLC production was the preparation of the oil and aqueous phases separately. Preliminary tests were conducted to determine the adequate volume of oil and aqueous phases, as well as the surfactant concentration. The oil phase consists of 1 mL of clove oil (CO) containing 50 or 100 mg of melted Gelucire 43/01 (GE) with 10 or 20 mg of Apocynin (APO), till the clear oily solution is produced. The aqueous phase (5 mL) contains 0.5 or 1% w/v of chitosan acetate (CH) and tween 80 (1% v/v), so the ratio of aqueous to oil phase is 5:1 respectively. Chitosan acetate was prepared by dissolving LMW-CH in 1% (v/v) acetic acid solution, then filtered by 0.45 µm membrane filters (EMD Millipore, Billerica, MA, USA)^41^. The aqueous phase was heated to the same temperature as the oil phase (45°C), and then both were mixed using a magnetic stirrer (Magnetic stirrers, Thermolyne Corporation, Dubuque, Iowa, USA), to form the pre-emulsion of pH 5. The pre-emulsion was then sonicated for 10 min at the following settings: (Amplitude: 90%, Timer: 10 min, Pulser: 1 s ON/ 1 s OFF, probe temperature: room temperature (25 ± 1 °C)), using a probe sonicator (4710 Series, Cole-Parmer Instrument Co., Chicago, USA)^42^. APO-loaded CO-based NSLCs were separated by centrifugation at 12,000 rpm for 90 min at − 4 °C (Cooling centrifuge, CE16-4X100RD, ACCULAB, USA), followed by washing with deionized water and freeze-drying under vacuum at − 80 °C (Freeze dryer, SIM FD8-8T, SIM International, USA). The supernatant would be saved for indirect determination of EE %^13,42,43^. Ultimately, the lyophilized NSLCs were stored for further evaluation at 4 °C. Plain CO-based NSLCs were exactly prepared as stated above, without adding APO to the formulation. Table 2 shows the composition of APO-loaded CO-based NSLC formulations.

**Table 2:** Formulations and CQAs values of APO loaded CO-based NSLCs as claimed by 2^3^ full factorial design.

Formula code	CPP			CQAs			
	XA	XB	XC	EE (%)	PS (nm)	PDI	ZP (mv)
F1	20	100	1	61.0 ± 3.8	319.7 ± 13.0	0.22 ± 0.01	+ 30.6 ± 0.9
F2	20	100	0.5	79.3 ± 0.9	214.8 ± 5.8	0.18 ± 0.01	+ 37.5 ± 1.1
F3	20	50	1	76.7 ± 1.1	1154.3 ± 76.7	0.59 ± 0.03	+ 34.6 ± 1.0
F4	20	50	0.5	76.0 ± 1.2	818.2 ± 31.1	0.58 ± 0.08	+ 26.7 ± 0.8
F5	10	100	1	50.2 ± 0.8	435.2 ± 39.7	0.30 ± 0.01	+ 28.4 ± 0.9
F6	10	100	0.5	55.2 ± 0.9	375.5 ± 29.9	0.25 ± 0.01	+ 27.6 ± 1.1
F7	10	50	1	36.4 ± 0.9	484.5 ± 30.2	0.25 ± 0.01	+ 24.7 ± 0.8
F8	10	50	0.5	51.0 ± 2.0	662.6 ± 13.2	0.34 ± 0.001	+ 23.9 ± 0.9

Each value represents the mean ± SD (n = 3).

1 mL of clove oil (CO) and 1% of Tween 80 were constant in all formulae.

*CPP* critical process parameters, *CQAs* critical quality attributes, *XA* APO amount (mg), *XB* GE amount (mg), *XC* CH concentration (%w/v), *PS* particle size (nm), *PDI* polydispersity index, *ZP* zeta potential (mv), *EE%* entrapment efficiency (%).

### Characterization of APO-loaded CO-based NSLCs

#### Entrapment efficiency (EE%)

The EE% of APO-loaded CO-based NSLCs was estimated indirectly based on measuring free APO amounts in the collected clear supernatants, after centrifugation at 12,000 rpm for 90 min^13^. Then, 0.1 mL of the clear supernatant was diluted to 100 mL with deionized water and quantified using a spectrophotometer (UV/VIS Spectro, double beam, Labomed Inc., USA). APO has a strong UV–Vis absorption, with two peaks at 275 and 307 nm^1^, while CO has only one λmax at 279 nm as reported before^2^. To avoid interference from CO, APO (EE%) was estimated at 307 nm against the comparable plain NSLCs’ supernatant as a blank^42^:

$$EE \%=\frac{total\, amount \,of\, the \,drug-amount \,of\, un\, entrapped\, drug\, in \,the\, supernantant}{total \,amount\, of\, the\, drug }\times 100.$$

#### Particle size (PS) and polydispersity index (PDI)

Malvern Zetasizer Nanoseries (Malvern Instruments Limited, UK) was used to assess the PS and PDI of the freshly assembled NSLCs after suitable dilution (0.1 mL of the generated dispersion was diluted to 10 mL with deionized water)^15^.

#### Zeta potential (ZP)

Measurement of surface charge was conducted to assess the colloidal stability. ZP of the freshly prepared samples of APO-loaded CO-based NSLCs were measured via Malvern Zetasizer Nanoseries (Malvern Instruments Limited, UK), adopting the Laser Doppler Anemometry (LDA) technique, after suitable dilution (0.1 mL of the generated dispersion was diluted to 10 mL with deionized water)^15^.

### Optimization of APO-loaded CO-based NSLCs

Design Expert® v.12 (Stat-Ease, Inc., Minneapolis, Minnesota, USA) was used to optimize APO-loaded CO-based NSLCs based on maximum EE%, ZP with minimal PS, and PDI.

### Characterization of the optimal APO-loaded CO-based NSLCs

#### Fourier transform infrared spectroscopy (FT-IR)

FT-IR spectrophotometer (Madison Instruments, Middleton, Wisconsin, USA), with scanning range 4000 to 500 cm^−1^, was used to characterize the chemical properties of APO, CO, GE, CH, their physical mixture corresponding to the optimal formula, the optimal CO-based NSLC without adding APO (plain optimal), and with adding APO (F2)^42^.

#### Differential scanning calorimetry (DSC)

DSC was carried out through a Differential scanning calorimeter (Perkin-Elmer 4, USA), standardized with indium (m.p = 156.6 °C, purity of 99.99%). Briefly, in airtight sealed aluminum pans, 4 mg of each sample (APO, CO, GE, CH, their physical mixture matching the optimized formula, the optimal CO-based NSLC without adding APO (plain optimal), and with adding APO (F2) were separately heated at a rate of 10 °C/min, over a temperature range of 30–400 °C and under continuous dry nitrogen purging at 20 mL/min^42^.

#### X-ray diffractometry (XRD)

XRD is a special method for examining any changes in the crystallinity of substances before and after formulation. Using a Diano X-ray diffractometer (USA) outfitted with Co-K radiation (45 kV, 9 mA, scanned from 3° to 50° at 2 angles), X-ray diffractograms of APO, CO, GE, CH, their physical mixture corresponding to the optimized formula, the optimal CO-based NSLC without adding APO (plain optimal), and with adding APO (F2), were obtained^42^.

#### Transmission electron microscopy (TEM)

The optimal formula (F2) was subjected to morphological analysis using a JEOL TEM (100 CX, Japan). One milliliter of the fresh lipid dispersion (F2) was diluted ten times with ultrapure water. Then it was sonicated for 5 min, dropped on a copper grid coated with Formvar (200 mesh, Science Services, Munich, Germany), and left to dry at room temperature before being imaged^44^.

### In vitro release study

APO diffusion from the optimal formula (F2) and the control aqueous solution of the free drug were both studied using modified vertical Franz diffusion cells^13,43^. The in vitro release assays were carried out in three distinct dissolving media (0.1 N HCl of pH 1.2, PB of pH 6.8, and PB of pH 7.4), simulating stomach, intestine, and blood respectively. At predetermined intervals from 0.5 to 12 h, samples of 2 mL were withdrawn and replaced with an equal volume of the release medium^13,43^. Three centimeter-diameter Franz diffusion cells were fixed in a shaking incubator (GFL Gesellschaft für Labortechnik, Burgwedel, Germany), and kept at a constant temperature (37 ± 0.5 °C) throughout the experiment. Spectra/PorTM cellulose membrane (MW cut off of 12,000–14,000 Da, Spectrum Medical Industries Inc., Los Angeles 90054, USA) was soaked in the release medium for 12 h before being firmly clipped between the donor and receptor compartments. One milliliter of the control aqueous solution (2.7 ± 0.2 mg/mL APO in distilled water), or of the optimal formula (F2), was injected into the donor compartment. The dialysis medium (100 mL) was added to the receptor compartment, which was then shaken at a speed of 100 rpm. To keep the sink condition throughout the experiment, withdrawn aliquots of the release medium (2 mL) were replaced with an equivalent volume of fresh media at regular intervals. The collected aliquots were filtered through a Millipore filter (0.45 µm, Berlin, Germany) and subjected to a UV–Vis spectrophotometer at 307 nm for drug concentration analysis after appropriate dilution. The cumulative APO released (%) was computed for each experiment in triplicate at each time point^13^.

### Kinetic analysis of the drug release data

The release data were fitted to various kinetic models, including zero-order, first-order model, Higuchi’s model, Korsmeyer-Peppas kinetic, and Weibull model^45,46^. Based on the lowermost Akaike information criterion (AIC) values and the greatest coefficient of determination (R^2^), the kinetic model reflecting the optimal kinetic release profile was chosen^42^.

### Stability study

APO-loaded CO-based NSLCs’ shelf life was assessed through a stability study. Samples of freshly prepared optimal formula (F2) were kept in tightly sealed glass bottles and stored at two different conditions (refrigerated (4 ± 1 °C) and ambient (25 °C ± 2 °C/60% ± 5% RH))^13,43^. The stability of the optimal formula was assessed in terms of EE%, PS, PDI, and ZP at zero time, and every month over the 3-month storage period^29^.

### Cell toxicity assay

Using the methyl thiazolyl tetrazolium (MTT) test, the cytotoxicity investigations of APO, CO, the optimum formula (F2), and its plain counterpart were conducted on human urinary bladder cancer cell line (T24, ATCC® HTB-4TM). Once the cells were taken from the American Type Culture Collection in Manassas, Virginia, they were cultivated using dulbecco’s modified eagle medium (DMEM), as claimed by previous reports^47^. Plain T24 cell line without sample treatment was used as a control group. Cells were then treated with various doses of each sample ranging from (2.5, 5, 10, 20, 40, 80, and 100 µg/mL) after attaining confluency in 96-well plates. The cells were incubated for 24 h at 37 °C with 5% CO_2_ and 90% relative humidity. The following equation was used to get the % cell viability^48^:

$$Cell\, viability \%= \frac{absorbance \,of \,treated\, cells}{absorbance\, of\, untreated\, cells\, (control)}\times 100.$$

Using GraphPad Prism® software (GraphPad Software, Inc., version 9.0.0, San Diego, California, USA), the cell viability (%) values were graphically plotted against their corresponding log concentrations. From the equation of the obtained best-fitted line, the concentration that caused inhibition of 50% cell viability (IC_50_) was calculated^49^.

### Evaluation of the prophylactic effect of the optimal APO-loaded CO-based NSLC (F2) against cyclophosphamide (CP) induced hemorrhagic cystitis (HC) in rats

#### Animals

Male Sprague–Dawley rats (225–250 g) were acclimatized for a week in the medical experimental research center (MERC, Mansoura University) animal house, under recommended temperature settings (20–25 °C), with a steady 12 h light/dark cycle and unlimited access to a standard diet as well as water. After receiving approval from the Faculty of Pharmacy Ethical Committee at Mansoura University in Egypt (Ethical approval Code: 2023-14), all experiments were conducted in obedience to the standards of “Principles of Laboratory Animal Care” (National Institute of Health Publication No. 85-23, revised 1985). The research was also conducted and reported in compliance with the ARRIVE (Animal Research: Reporting of In vivo Experiments) standards.

#### Experimental protocol

To assess the prophylactic action of the optimal formula (F2) against CP-induced HC in rats, 36 rats were equally categorized into six groups (six rats per group) as follows:

- Group I: (normal control) rats received only 2 mL of normal saline (NS).
- Group II: (positive control) rats received CP (150 mg/kg/i.p).
- Group III: (Mesna group) rats received Mesna (90 mg/kg).
- Group IV: rats received 2 mL pure APO suspension in 0.5% CMC.
- Group V: rats received 2 mL plain optimal CO-based NSLCs.
- Group VI: rats received 2 mL F2-NSLCs.

In such a regimen, all groups received oral pretreatment via gastric gavage for 7 days before induction of HC. On the eighth day, HC was induced in all groups, except for the normal control one, by i.p injection of CP (150 mg/kg)^50^. Special treatment for Group III rats, they were injected with a total dose (90 mg/kg, i.p) of Mesna in three equal doses (each of 30 mg/kg). The first injection was given 20 min before the CP injection, and the subsequent injections were given 4 and 8 h later, respectively^51^. All animals were sacrificed on the ninth day by i.p injection of ketamine HCL (80 mg/kg) and xylazine HCL (10 mg/kg)^52^. Rats’ bladders were evacuated from urine. Then they were dissected for further assessment after being macroscopically examined.

#### Macroscopic examination of the bladder

According to pre-established criteria, inspected bladders were macroscopically checked for weight, visible edema, and hemorrhage. Edema was rated as severe (3+), moderate (2+), mild (1+), between normal and moderate, and none (0) as normal, conferring their visual presence internally and externally in the bladder wall. The following criteria were used to grade the hemorrhage: intravesical clots (3+), mucosal hematomas (2+), telangiectasia (1+), and normal (0+). The scores gained from observing each animal’s bladder were summed to provide the overall score for each group^53^.

#### Histopathological evaluation

The following steps were used to prepare the bladder samples: washing with sterile water, lengthwise division, fixation in buffered formalin (10%), blocking with paraffin, sectioning into 5 µm sections, and hematoxylin and eosin (HE) staining. These slides were analyzed using a Nikon Eclipse Ci connected Kameram® Digital Image Analyse System under a light microscope. According to the next criteria (desquamation, epithelial hyperplasia, epithelial degeneration, propria vascularization, edema, inflammatory alterations, basement membrane damage, and hemorrhage) slides were classified as severe (3), moderate (2), mild (1), and normal (0)^51^.

### Immunohistochemical (IHC) assessments of nuclear factor kappa B (NF-κB) and cyclooxygenase-2 (COX-2)

Bladder tissue slices were prepared as stated in the manufacturer’s instructions (Thermo Fisher Scientific, Waltham, MA, USA). Then sections were washed with PBS (pH 7.4), before being incubated with the primary antibodies against NF-κB (1:100) and COX-2 (1:50) in 1% bovine serum albumin (BSA) contained in PBs, overnight at 4 °C. PBs were used as a negative control in place of the main antibody. Immunostaining was applied to the sections, and the score grades were as follows: strong staining = 3, moderate staining = 2, no mild staining = 1, and no staining = 0^54^.

### Assessment of oxidative stress and anti-oxidant parameters

Plasma samples were collected using EDTA as an anticoagulant, centrifuged for 15 min at 1000×*g* within 30 min of collection, and assayed immediately. Immunoassay kits were used to measure the plasma concentration of glutathione (GSH), malondialdehyde content (MDA), total nitric oxide (NO), and superoxide dismutase levels (SOD). Inspected sample volumes and dilutions were determined according to each assay requirement. Measurements were performed in triplicates for all samples^50^.

### Statistical analysis

2^3^ full factorial design was statistically analyzed using one-way analysis of variance (ANOVA) by Design Expert® v.12 (Stat-Ease, Inc., Minneapolis, Minnesota, USA). The validity and appropriateness of the regression models were statistically significant when adjusted coefficients of determination (adjusted R^2^) between 0.8 and 1.0 and *F-*values (*p* < 0.05)^42^. The mean standard deviation (SD), (n = 3), and mean standard error of the mean (SEM), (n = 6), respectively, were used to represent the experimental results from both in vitro and in vivo studies. A computer program (GraphPad Prism Software Inc., San Diego, CA, version 9.3.1) was used to do the statistical analysis. One-way analysis of variance (ANOVA) was used to analyze the parametric data, and then the Tukey–Kramer test for multiple comparisons. The non-parametric data, on the contrary, were analyzed using the Kruskal-Walli’s test, followed by the Dunn multiple comparison test for both the IHC score and histology. The *p* values were statistically significant at the cutoff of *p* < 0.05.

### Ethical approval

The study protocol was reviewed and accepted by the ethical committee of the Faculty of Pharmacy, Mansoura University, Mansoura, Egypt, following the “Principles of Laboratory Animal Care, National Materials Institute of Health Publication (No. 85-23, revised 1985)”, (Ethical Approval Code 2023-14).

## Result and discussion

### Preparation, characterization, and optimization of APO-loaded CO-based NSLCs by DOE

Table 1 represents the 2^3^ full factorial design and CPPs levels, while the outcomes of CQAs for various formulations are described in Table 2. The polynomial-coded equations provided a summary of each CPP’s impact on EE%, PS, PDI, and ZP. A positive value preceding a factor implies that response rises with this factor, whereas a negative value suggests the opposite^55^.

### Entrapment efficiency (EE%)

The EE% of APO-loaded CO-based NSLCs ranged from 36.4 ± 0.9 to 79.3 ± 0.9%. The obtained polynomial equation representing the regression models for this CQA is as follows:

$${\text{EE }}\% = { 6}0.{7625 } + { 12}.{\text{52XA }} + \, 0.{\text{71XB }} - { 4}.{\text{63XC }} - { 3}.{8}0{\text{XAB }} + \, 0.{\text{24XAC }} - { 1}.{\text{17XBC }} - { 3}.{\text{57XABC}},$$

where F = 224.73, *p* < 0.0001, CV % = 2.93 and adjusted R^2^ = 0.9855.

A thorough examination of the previous equation reveals that the most prominent effect on EE% was for APO (XA) amount, as demonstrated by the higher coefficient of XA (12.52).

The impact of both APO (XA) and GE (XB) on the EE% is highly influencing, as seen in Table 2. F2 (20 mg APO, 100 mg GE, and 0.5% CH) has an EE% value of 79.3 ± 0.9% and has high levels of APO (20 mg) and GE (100 mg). This value dropped to 51.0 ± 2.0% for F8 (10 mg APO, 50 mg GE, and 0.5% CH) upon decreasing both CPP (APO & GE), to lower levels, while keeping CH (XC) constant at the low level (0.5%). The apparent explanation for these results may be due to the highly viscous lipid (GE), which prevents drug escape from the core of the internal oil phase^13^. On the other hand, an increase in CH concentration (XC) to 1% as in F1 (20 mg CH, 100GE, and 1% CH) & F7 (10 mg APO, 50 mg GE, and 1% CH) resulted in a subsequent decrease in EE%. This may be explained by the increasing concentration of CH leads to an increase in viscosity and gel formation, which hindered the encapsulation of APO^56^.

The model equation for EE% was applied to construct several graphical representations, which made it much easier to grasp how CPPs affected CQAs. The variations in the contour and 3D surface plots of the aforementioned CQAs versus the two CPPs, XB (GE) and XC (CH), are shown in Fig. S1A–D, respectively. The other CPP, XA (APO), is left fixed at its low and high values.

According to the contour plots, the CQA (EE%) was at its utmost with high concentrations of APO (XA), GE (XB), and low concentrations of CH (XC). This indicates that the production of NSLCs with high EE% (F2) is encouraged by these values. The most likely reason is that during the production of the NSLCs, the high concentration of GE (XB) provided additional space to accommodate the medication in excess amounts^13^. High lipid content also reduces the quantity of APO that escapes into the external aqueous phase, and increasing EE%. Contrarily, increasing CH concentration reduces drug entrapment^56^.

### Particle size (PS) and polydispersity index (PDI)

PS and PDI are two crucial touchstones for NPs because they affect how drugs are absorbed, distributed, and subsequently bioavailable^57^. Wu et al. reported that smaller NSLCs (> 250 nm) were able to improve the absorption of poorly soluble drugs compared to bigger ones^58^. As detailed in Table 2, all formulations exhibited PS ranging from 214.8 ± 5.8 to 1154.3 ± 76.7 nm and PDI values ranging from 0.18 ± 0.01 to 0.59 ± 0.03. The small values of PDI describe the narrow-average size distribution of the NSLCs formed^55^.

Making predictions about the response for the specified levels of each factor is possible using the coded equation. By default, the high factor levels are coded as + 1 and the low ones are coded as − 1. The coded equation is convenient for recognizing the expected effect of each factor by matching the factor coefficients. The following polynomial equations that describe the regression model for PS and PDI are:

$${\text{PS}} = { 558}.{15 } + { 68}.{\text{65XA }} - { 221}.{8}0{\text{XB }} + { 4}0.{\text{33XC }} - { 137}.{\text{69XAB }} + { 69}.{\text{91XAC }} + \, 0.{\text{84XBC }} - { 58}.{\text{62XABC}},$$

where F = 213.81, *p* < 0.0001, CV% = 6.53 and adjusted R^2^ = 0.9848,

$${\text{PDI}} = \, 0.{34 } + \, 0.0{\text{53XA }} - \, 0.{\text{1XB }} + {\text{ XC }} - \, 0.0{\text{9XAB }} + \, 0.0{\text{1XAC }} + \, 0.0{\text{2XBC }} - \, 0.0{\text{1XABC}},$$

where F = 1093.55, *p* < 0.0001, CV% = 2.45 and adjusted R^2^ = 0.9970.

These equations disclose that all CPPs and their interactions are significant towards PS and PDI. APO amount (XA) and CH concentration (XC) have the most prominent impact on increasing PS and PDI if their levels are kept high. Meanwhile, GE amount (XB) demonstrated a strong negative effect on both CQAs. Tcholakova et al*.* have reported that the size of nanoparticles varies with the surfactant content, as it adsorbed on the surface of NSLCs forming a monolayer^59^. This adsorption depends on the chemical nature of the lipid matrix and the surfactant used. GE 43/01 acts as a hydrophobic stabilizer, it forms a monolayer on the surface of APO-loaded NSLCs through van der Waal’s interaction between mono, diglycerides, and polyethylene glycol esters of fatty acids present in its structure, leading to PS reduction^30,60^.

From the above equation of PS, the interaction between APO and GE (+ XAB) results in a decreased PS (Table 2) regardless of the concentration of CH (XC). As indicated in F1, F2 high amounts of APO (20 mg), and GE (100 mg) were used compared to F7, F8 (10 mg APO, and 50 mg GE), the resulting PS was about 319.7 nm, and 214.8 nm, versus 484.5 nm, and 662.6 nm respectively. On the other hand, in F3 and F4 (20 mg APO, and 50 mg GE), large-sized particles were formed (1154.3 nm and 818.2 nm, respectively). This may be attributed to GE; which makes the lipid phase more able to dissolve more APO, and hence small particles could be obtained. At low levels of GE, the available lipid molecules would not be sufficient to cover and stabilize the high population of the formed NSLCs leading to particle coalescence, and the formation of particles with bigger sizes^61^.

To make it much simpler to understand how CPPs affect CQAs, some graphical representations based on the model equation for PS were created. Figure S2a–d depicts contour and 3D plots, respectively, of variations in the aforementioned CQAs versus changes in the two CPPs, GE (XB) and CH (XC), in the analyzed range, while APO (XA) is kept constant at its low and high levels.

### Zeta potential (ZP)

Depending on the chemistry of the particles and the strength of the attraction between like-charged particles in the dispersion medium, NSLCs can have either positive or negative polarity. In the dispersion medium, NPs with ZP more than + 30 mV or less than − 30 mV are considered extremely stable^62^. All assembled formulae’s ZP values were uniformly positive and ranged from + 23.9 ± 0.9 to + 37.5 ± 1.1 mV (Table 2). These positive charges originated from CH’s vacant amine groups. Singular particles can arise because of increased repelling force caused by increasing positive charge^55^. Positive ZP is necessary to increase drug absorption by boosting mucoadhesion and drug bioavailability^13,63^. The Tween 80 surface coating of NPs decreases the ZP by sterically stabilizing the particles’ electrophoretic mobility^64^. Herein, the ZP of F2 is + 37.5 ± 1.1 mV, which is sufficient in the presence of tween 80 to fully stabilize the system. The regression model for ZP is well-defined through the following equation:

$${\text{ZP}} = { 29}.{2667 } + { 3}.0{\text{8XA }} + { 1}.{\text{77XB }} + \, 0.{\text{34XC }} - \, 0.0{\text{75XAB }} - \, 0.0{\text{91XAC }} - { 1}.{\text{85XBC }} - { 1}.{\text{85XABC}},$$

where F = 68.89, *p* < 0.0001, CV% = 3.38 and adjusted R^2^ = 0.9538.

All CPPs have positive coefficients towards ZP with relatively small numerical values, according to the polynomial equation. However, the coefficients of each of these interactions are all negative towards ZP.

### Optimization of APO-loaded CO-based NSLCs

An optimized formula was proposed once the ideal outcome for each independent variable was determined. F2, with (+ XA, + XB, − XC), was chosen as the optimal formula based on the previously provided optimization criteria (maximum EE%, ZP with minimal PS, and PDI). The compatibility of the optimized formula’s component (F2) will be further characterized using FT-IR, DSC, and XRD, followed by surface morphology assessments using TEM, in vitro drug release, stability, and cytotoxicity, as well as in vivo evaluation on a model of hemorrhagic cystitis.

### Characterization of the optimal APO-loaded CO-based NSLC formula (F2)

#### Fourier transform infrared spectroscopy (FT-IR)

**Figure 1:**
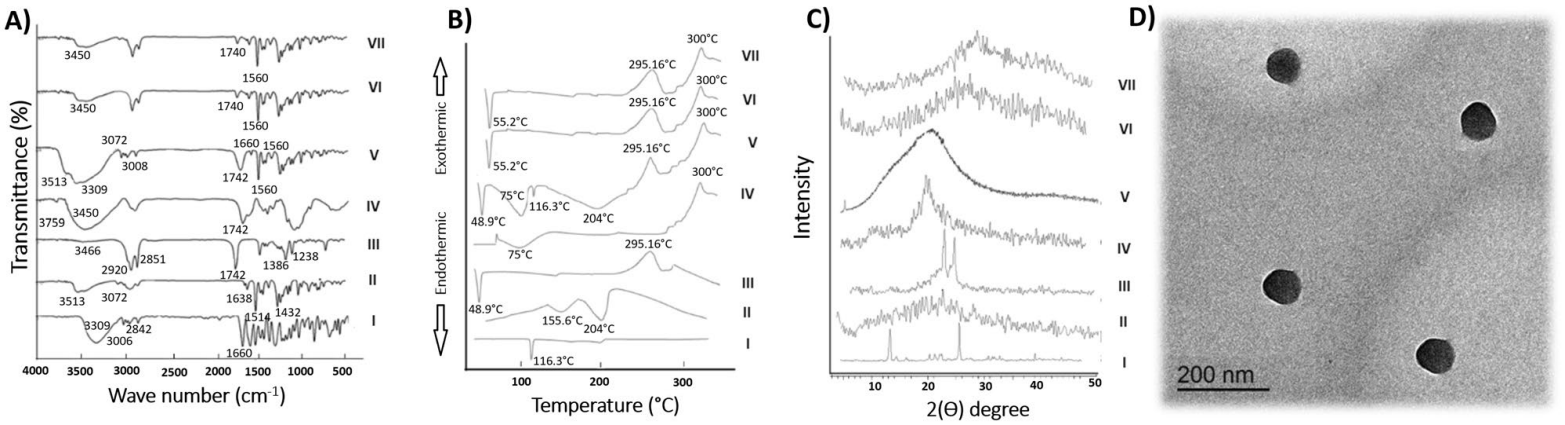
(**A**) FT-IR spectra of (**I**) APO, (**II**) CO, (**III**) GE, (**IV**) CH, (**V**) physical mixture, (**VI**) plain optimal formula, and (**VII**) optimal formula (F2). (**B**) DSC spectra of (**I**) APO, (**II**) CO, (**III**) GE, (**IV**) CH, (**V**) physical mixture, (**VI**) plain optimal formula, and (**VII**) optimal formula (F2). (**C**) XRD spectra of (**I**) APO, (**II**) CO, (**III**) GE, (**IV**) CH, (**V**) physical mixture, (**VI**) plain optimal formula and (**VII**) optimal formula (F2). (**D**) TEM image of APO loaded CO-based NSLCs optimal formula (F2).

Figure 1A denotes the FT-IR spectra of the optimal formula (F2) and its constituents. As indicated in Fig. I, infrared peaks at 3309, 3006, 2842, and 1660 cm^−1^, respectively, represent the functional phenolic OH, aromatic hydrogen, alkane carbon-hydrogen, and ketone C=O conjugated bonds of APO^13^. FT-IR spectrum of CO (Fig. II), showed a characteristic peak at 3072 due to O–H stretching. Peaks corresponding to eugenol were typically denoted at 1638 and 1514 cm^−1^, whereas those that appeared at 1432 cm^−1^ were due to C–C stretching vibrations of the phenyl ring^65^. The typical peaks of GE (43/01) were at 3466 cm^−1^ (O–H stretching), 2920 and 2851 cm^−1^ (C–H stretching), 1742 cm^−1^ (C=C stretching), and 1238–1386 cm^−1^ (C–O–C stretching), as depicted in Fig. III^66^. Two distinctive bands at 1659 and 1596 cm^−1^ were seen in the CH spectra (Fig. IV) due to the carbonyl stretching vibrations of the secondary and primary amide groups, respectively. N–H, O–H stretching, and intramolecular hydrogen bonding were all present as a strong band in the area of 3450 cm^−1^. Prior reports of homologous bands exist^55^. The individual ingredient bands could be seen in the spectrum of the physical mixture (Fig. V), but the APO bands were either diminished or completely lost because of the dilution effect. The plain optimal formula, as well as the corresponding medicated one (F2), showed representative peaks at 3450, 1560, and 1740 cm^−1^, corresponding to CH and GE, respectively. Notably, figure prints of both APO and CO disappeared, indicating the successful incorporation of the drug in the core of the optimal formula (Fig. VI,VII).

#### Differential scanning calorimetry (DSC)

The DSC spectra of the optimal formula (F2), as well as its constituents, were represented in Fig. 1B. APO showed a melting endotherm at 116.3 °C (Fig. I), indicating pure APO’s crystalline nature^13^. At 155.6 °C and 204 °C, pure CO (Fig. II) displayed distinct endothermic reactions that may be attributed to boiling and evaporation phenomena^15^. Figure III shows the DSC curve of GE 43/01, with an endothermic peak at 48.9 °C due to GE melting, and an exothermic one at 295.16 °C^60^. Due to polymer dehydration and breakdown of CH (Fig. IV), it displayed a broad endothermic peak at 75 °C and an exothermic one at about 300 °C^55^. The DSC thermogram of the physical mixture showed all the previously indicated peaks of GE and CH at their appropriate locations, with a noticeable reduction of the peak of APO due to the diluting effect (Fig. 5). The DSC of both the plain optimal formula and the medicated optimal one (F2) matched each other. The APO peak disappeared from the medicated nanoparticles thermogram, suggesting drug entrapment in the nanostructured lipid particles. Both spectra of plain and medicated nanoparticles showed a small shift in the GE peak, with a reduction of both CO and CH endothermic peaks (Figs. VI,VII). These results with FT-IR ones coincide with the incorporation of APO within the NSLC system.

#### X-ray diffractometry (XRD)

XRD patterns represent the structural nature, either crystalline or amorphous, of the optimal formula (F2), besides their components (Fig. 1C). APO’s crystallinity (Fig. I), was demonstrated by prominent diffraction peaks at 2θ of 13.125°, 22.611°, and 26.413°^13^. Since CO is naturally viscous, its XRD pattern only displays one wide peak at 11.45°–13.35° (Fig. II), with no other positions exhibiting strong spikes^65^. On the other hand, GE’s XRD diffractograms (Fig. III) exhibit moderately strong peaks at 21.32° and 23.55° (2θ)^67^. The crystalline structure of CH powder was shown by a distinct peak at a 2θ diffraction angle of 20° (Fig. IV). The distinctive peaks associated with the aforementioned elements were also seen in the pattern created by the physical mixture (Fig. V), but those associated with APO were diminished due to dilution. The plain optimal formula diffractogram (Fig. VI), together with the medicated optimal one (Fig. VII), were nearly similar, as both showed a notable disappearance of APO peaks. This may be due to the loss of APO crystalline structure, APO encapsulation, and formation of amorphous NSLCs in F2^42,68^.

#### Transmission electron microscopy (TEM)

TEM is a method of probing the structure of nanosystems^13^. The TEM image of the optimal formula (F2), in Fig. 1D, shows that the designed NSLCs have a spherical shape with a CO-based core wrapping APO along with GE and a shell of CH. The PS of the optimal formula (F2) determined by zetasizer was nearly similar to that measured by TEM.

### In vitro drug release study

**Figure 2:**
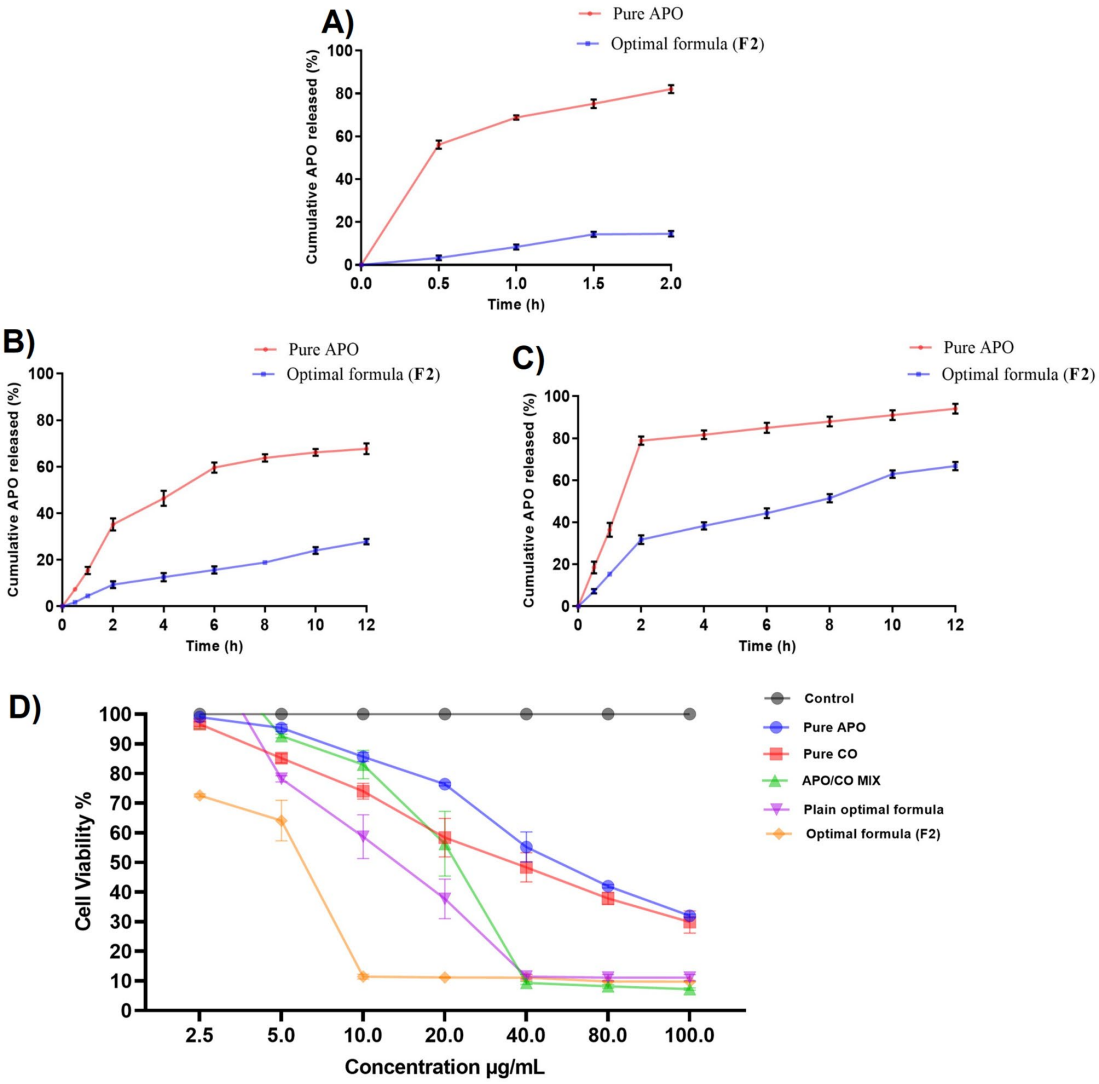
The in vitro (**A**) release pattern of APO from aqueous solution in comparison with the optimal formula (F2) at three different pH media: (**I**) pH 1.2, (**II**) pH 6.8, and (**III**) pH 7.4. (**B**) cytotoxicity screening for pure APO, pure CO, APO/CO mixture, plain optimal formula, and the optimal formula (F2) in addition to the control group (non-treated cells) against the T24 cell line. Each point represents the mean ± SD (n = 3).

Since free APO is an amphoteric molecule, it dissolves in both acidic and basic solutions^13,43^. At pH 1.2 and pH 7.4, around 80% of the free APO was released in the first 2 h (Fig. 2A,C, respectively). This might be simply because of water molecules (H_2_O) that form a hydrogen bond in the acidic media with the drug’s phenolic (OH) group^13,43,54^. At basic pH, the drug’s phenolic OH group was readily ionized. Hence, interactions with the Na+ or K+ cations of the PB may take place, as well as hydrogen bond formation with water molecules. All of these may cause total diffusion of free APO^13^. On the other hand, at a nearly neutral pH of 6.8, the percentage cumulative drug release from free APO reached about 30% after 2 h. The maximum percent of drug release was after the first 6 h (60%); then the release rate was significantly reduced to 12% over the next 6 h (Fig. 2B). This behavior can be attributed to a reduction in the ionization of APO at a neutral pH, causing a decrease in its solubility^13^. APO in vitro release data from the optimal F2-NSLCs revealed no burst effect at any of the different release media, otherwise, the release was sustained for 12 h, as shown in Fig. 2A–C. Distinguishably, the percentage cumulative drug release of APO from the optimal formula (F2), after 2 h at pH 6.8, was only 9.3%, versus 14.5% at pH 1.2, and 31.8% at pH 7.4. The hydrophobicity plus the viscosity of the lipid matrix (GE) plays the main role in this sustained behavior^69,70^. On the other hand, the protective coating of NSLCs with CH also provides a physical shield to the drug from being diffused^31^. It all makes the NSLCs an appropriate platform to develop sustained-release drug delivery systems for APO.

### Kinetic analysis of the drug release data

The pH of the medium had an impact on the kinetic analysis of APO release, as shown in Table S1. Interestingly, the release kinetics of the optimal formula (F2) were better described by zero and first-order models at pH 1.2 and 6.8, respectively, whereas Higuchi’s model with a non-Fickian diffusion mechanism was a better fit at pH 7.4. Since n > 0.5 and β > 0.75 for the Korsmeyer-Peppas and Weibull models, respectively, it was concluded via complementary analysis that APO release from the optimal formula (F2) at pH 7.4 was regulated by a Non-Fickian diffusion mechanism^42^.

### Stability study

Table 3 details the data for the stability characteristics of the optimal APO-loaded CO-based NSLCs (F2). The ANOVA findings revealed a negligible fluctuation in EE%, PS, PDI, and ZP during the refrigerated storage period. On the other hand, after being stored at room temperature, there was a noticeable increase in PS and PDI, as well as a decrease in EE% and ZP. These results demonstrate the stability of the optimal formula (F2) after three months of refrigeration, which is proven by a consistent size range and homogeneous distribution^13,29^.

**Table 3:** Stability assessment parameters of the optimal formula (F2) following storage at different temperature conditions (see Table 2 for F2 composition).

Temp/humidity	Month	EE%	PS (nm)	PDI	ZP (mv)
Refrigerated conditions (4 ± 1 °C)	0	79.3 ± 0.9	214.8 ± 5.8	0.18 ± 0.01	37.5 ± 1.1
	1	79.1 ± 0.5	215.3 ± 1.3	0.18 ± 0.05	37.6 ± 1.1
	2	78.5 ± 0.3	215.7 ± 1.1	0.18 ± 0.07	37.6 ± 0.4
	3	78.3 ± 0.1	216.1 ± 0.9	0.18 ± 0.08	37.6 ± 0.5
Ambient conditions (25 °C ± 2 °C/60% ± 5% RH)	0	79.3 ± 0.9	214.8 ± 5.8	0.18 ± 0.01	37.5 ± 1.1
	1	75.3 ± 1.3*	223.2 ± 2.1*	0.22 ± 0.07*	29.8 ± 1.2*
	2	70.1 ± 1.2*	233.6 ± 3.2*	0.34 ± 0.05*	25.3 ± 1.1*
	3	63.9 ± 1.6*	245.3 ± 2.41*	0.36 ± 0.06*	23.9 ± 1.5*

Each value represents the mean ± SD (n = 3).

*PS* particle size (nm), *PDI* polydispersity index, *ZP* zeta potential (mv), *EE%* entrapment efficiency (%).

*Significant at *p* < 0.05 monthly vs. initial.

### Cell toxicity assay

As depicted in Fig. 2D, increasing the sample concentration resulted in declining cell viability (%) in a dose-dependent manner. The highest effect was with a concentration of 100 µg/mL. Careful examination of cell viability (%) results shows that CO was more cytotoxic than APO when each was used alone at the same concentration. The cytotoxic effect of CO may be attributed to its eugenol content^71^. Formulation of CO-based NSLCs (plain optimal formula) ameliorated its cytotoxicity; by their tunable size and surface properties, which boost CO cellular uptake^72,73^. APO/CO mix showed good synergism in the inhibition of cell viability (%) at concentrations lower than 40 µg/mL (Fig. 2D). This activity amplified after the formulation of the optimal formula (F2), which yielded a significant reduction in cell viability (%) at lower concentrations (≥ 10 µg/mL). The cytotoxicity findings were consistent with the calculated IC_50_ results (Table 4). It was clear that CO was highly cytotoxic compared to APO (IC_50_ = 35.7 ± 1.9 for CO vs. 73.9 ± 2.5 µg/mL for APO); meanwhile, a mixture of both (APO/CO mix), has a synergistic effect (IC50 = 19.2 ± 1.3 µg/mL). The Plain optimal formula (CO only) showed an IC50 value of 12.2 ± 1.8 µg/mL compared to CO alone (35.7 ± 1.9 µg/mL) while adding APO reduced the IC50 to 5.8 ± 1.3 µg/mL (Table 4). The remarkable hike in cytotoxicity of the optimal formula (F2) was attributed to increasing both CO and APO’s cellular uptake and stability via the NSLC’s surface properties and tunable size, besides p-glycoprotein inhibition effects and muco-adhesion of CH particularly^74–76^.

**Table 4:** The in vitro cytotoxicity screening was computed as the 50% inhibitory concentration values (IC_50_) against the T24 cell line after 24 24-h incubation period.

Formula	IC_50_ μg/mL
Pure APO	73.9 ± 2.5
Pure CO	35.7 ± 1.9*
APO/CO mix	19.2 ± 1.3*^#^
Plain optimal formula	12.2 ± 1.8*^#$$^
Optimal formula (F2)	5.8 ± 1.3*^#$^^

Each value represents the mean ± SD (n = 3).

Statistical significance is indicated as: *p < 0.05 vs pure APO, ^#^*p* < 0.05 vs pure CO, ^$^*p* < 0.05, ^$$^*p* < 0.01 vs APO/CO mix, ^*p* < 0.05 vs plain optimal formula.

### CP-induced HC in rats’ model

#### Macroscopic examination

Cystitis observed 24 h after CP administration (Fig. S3) was characterized macroscopically by an increase in bladder weight, severe edema, and marked hemorrhage with mucosal hematomas, as well as intravesical clots. As illustrated in Fig. S4b,c, the positive control group achieved a score of 3, which was significantly different from the normal control one (score of 0) for edema and hemorrhage. CP evoked a rise in bladder weight, which was significantly inhibited by pretreatment of rats with 3 doses of Mesna, as well as oral pretreatment with the optimal formula (F2) or the corresponding plain one (Fig. S4a). Macroscopically, there was no significant difference between the effects of these 3 different strategies. Pure APO did not prevent the increase in bladder weight or other macroscopic changes. In brief, from the macroscopic results, pretreatment with Mesna alone or the plain optimal formula (CO only) as well as the optimal one (F2), but not APO alone, significantly reduced the intensity of cystitis as presented in Fig. S4a–c.

#### Histopathological evaluation

**Figure 3:**
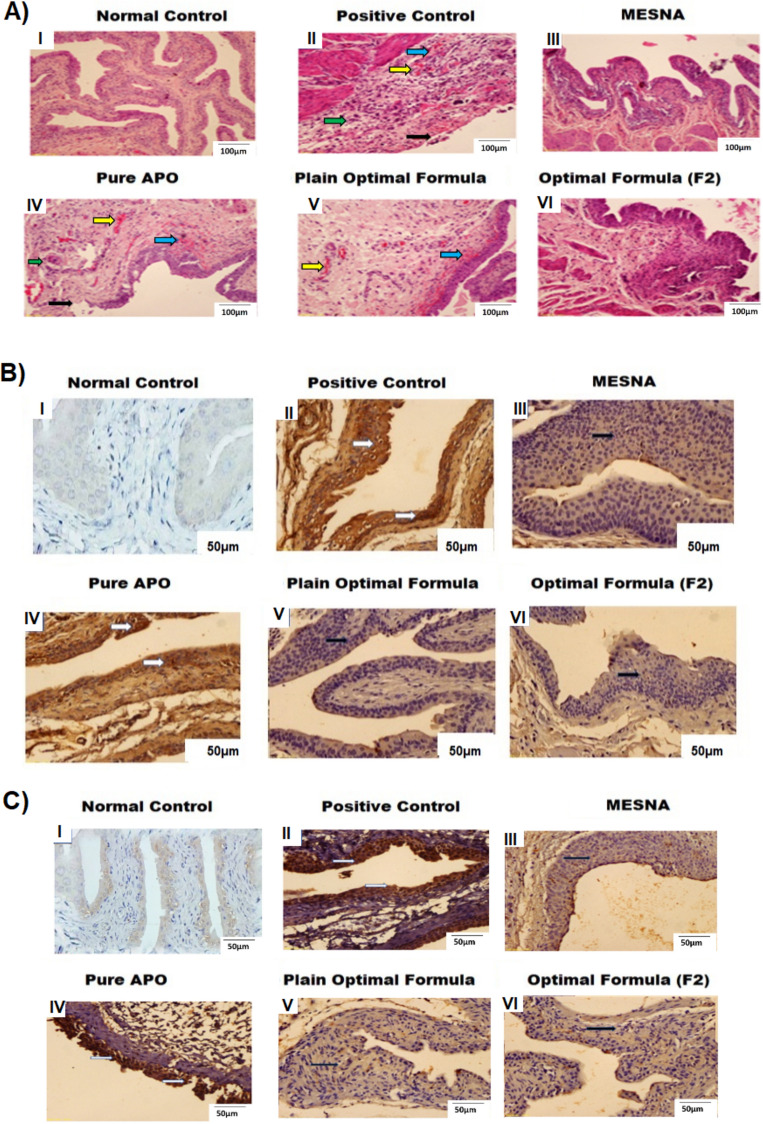
Representative microphotographs of (**A**) Rat’s bladder sections, H&E stained, from all experimental groups. Black arrows point to mucosal atrophy and ulceration. Yellow arrows point to capillary congestion. Green arrows point to stromal reactions. Blue arrowheads point to interstitial edema and hemorrhage. Low magnification (X: 100, bar: 100 µm). (**B**) Immune-stained bladder sections against NF-κB in all experimental groups. Black arrows point to negative IHC staining. White arrows point to positive or brown cytoplasmic expression, high magnification (X: 200, bar: 50 µm). (**C**) Immune-stained bladder sections against COX-2 in all experimental groups. Black arrows point to negative IHC staining. White arrows point to positive or brown cytoplasmic expression, high magnification (X: 200, bar: 50 µm).

Bladder sections microphotographs, from all experimental groups, have been stained with H&E, are shown in Fig. 3A. The sections from the normal control group revealed normal urothelium, lamina propria and musclaris propria (Fig. I). In contrast, the CP-induced HC group (positive control) showed extensive mucosal lining atrophy, congested capillaries of lamina propria, interstitial hemorrhage, edema in addition to stromal reactions; all of marked degree (score of 3) (Fig. II). Pretreatment with Mesna abolished these alterations (Fig. III). However, similar lesions as in the aforementioned positive control group were observed in groups administered the oral pretreatment regimen with pure APO (Fig. IV). Intriguingly, the deleterious interstitial cystitis manifestations in rats were attenuated by pretreatment with CO-based NSLCs (plain optimal formula), with a significant reduction in the inflammatory conditions (Fig. V). Undeniably, the most distinct uroprotective efficacy against CP-induced HC was implemented to groups pretreated with the optimal formula (F2), where cystitis manifestations were hardly recognized (Fig. VI).

### Immunohistochemical (IHC) assessment of nuclear factor (NF-κB) and cyclooxygenase-2 expression (COX-2)

One of the vital pathogenic factors in HC is an exaggerated response of inflammation due to the production of reactive oxygen species (ROS) following CP injection^77^. The pivotal nuclear transcription factor NF-κB plays a crucial role in inflammatory responses, amplifying pro-inflammatory cytokine production, promoting nitric oxide synthase (iNOS) expression, and enhancing nitric oxide (NO) as well as COX-2 expression. Inhibiting the NF-κB and COX-2 signaling pathways is essential for alleviating bladder injury induced by CP in rats^78–82^. To confirm the uroprotective effect of the optimal formula (F2), IHC assessments of expression levels of both NF-κB and COX-2 were performed.

Figure 3B,C show IHC analyses of the expressions of NF-κB and COX-2 in the bladder tissues, respectively. Negative staining was seen against both proteins in the normal control rats’ group. In contrast, in the bladder sections of the CP-induced HC group (positive control), the immunoreactivity against such proteins was identified as a strong positive brown cytoplasmic expression. Groups that had received prophylactic treatment with Mesna did not exhibit such immunoreactivities. In contrast, albeit slightly diminished, immunoreactivities were still seen in cases of pure APO administration. It is interesting to note that pretreatment with both the optimal formula (F2), which contains both APO and CO and the plain optimal, which contains CO only, distinctively reduced the intense positive cytoplasmic expressions for both (NF-κB and COX-2).

**Figure 4:**
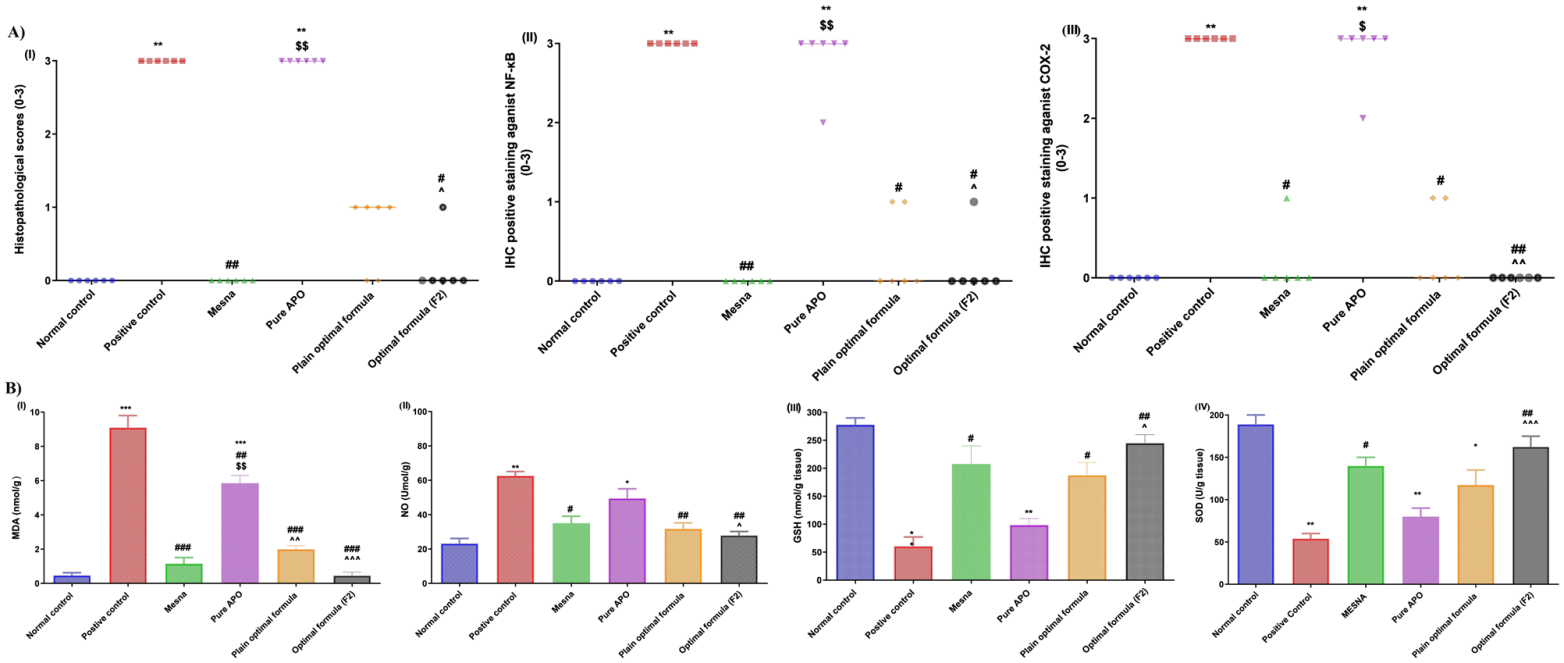
(**A**) Statistical analysis of histopathological score (A) and IHC scores of NF-κB (B) and COX-2 (C) in bladder sections of all experimental groups (n = 6). Statistical significances of rat groups are indicated as: **p < 0.01 vs normal control group, ^#^p < 0.05, ^##^p < 0.01 vs positive control group, ^$^p < 0.05, ^$$^p < 0.01 vs Mesna group and ^p < 0.05, ^^p < 0.01 vs pure APO group. Kruskal–Wallis’s test (nonparametric test) was applied followed by the Dunn multiple comparison test. (**B**) Influence of oral pretreatment with the optimal formula (F2) on CP-induced HC investigated changes in oxidative stress biomarkers: (A) MDA, (B) NO, (C) GSH, and (D) SOD in comparison with different experimental groups. Data are expressed as the mean ± SEM (n = 6). Statistical significances of rat groups are indicated as: *p < 0.05, **p < 0.01, **p < 0.001 vs normal control group, ^#^p < 0.05, ^##^p < 0.01, ^###^p < 0.001 vs positive control group, and ^p < 0.05, ^p < 0.01, ^^^p < 0.001 vs pure APO group. One-way analysis of variance (ANOVA) (parametric test) was applied followed by the Tukey–Kramer multiple comparison test.

Figure 4A (a–c, respectively) shows statistical analysis of the histopathological and IHC scores of NF-κB and COX-2 positive staining expression levels**.** The findings showed that the histopathological and IHC scores of the CP-induced HC group were significantly higher than those in the normal control group. In contrast, oral pretreatment with the optimal formula (F2) drastically reduced the histopathological and IHC scores. The successful consolidation of APO, CO, and GE within NSLCs wrapped by CH may be responsible for such downregulation of NF-κB and COX-2 protein expression levels; that was provoked by injection of CP^13,83,84^.

### Assessment of oxidative stress and anti-oxidant parameters

ROS levels in bladder tissues significantly intensify after treatment with CP, leading to rising MDA and NO levels, with exhaustion of intracellular endogenous supplies of anti-oxidants like GSH, and embarrassment of SOD level. Due to these serious side effects, the clinical use of CP is confined^51^.

In the current study, we experimentally tested whether pre-treatment with APO-loaded CO-based NSLCs could protect against CP-induced injury. As depicted in Fig. 4B (I–IV, respectively), the levels of the oxidative stress markers MDA, NO, GSH, and SOD in the bladder tissue were assessed. Significant (*p* < 0.05) elevation in the levels of both MDA and NO of the CP-induced HC group was detected, along with depletion of GSH and SOD levels compared to the normal control group. Notably, prophylactic administration of injectable Mesna, before and after CP injection, hinders the exponential increase in MDA and NO levels and sudden depletion of endogenous antioxidants (GSH and SOD)^85^. This outcome stems from the ability of Mesna to bind and detoxify acrolein, which in turn inhibits its oxidative activity and potentially limits CP-associated toxicities^37^. Undoubtedly, the optimal formula (F2) pretreated group had significantly (*p* < 0.05) decreased plasma levels of MDA and NO, and so far, retained nearly normal levels of GSH and SOD. Such impact could be related to the established anti-lipid peroxidative activity of APO, which looks to be augmented after loading on CO-based NSLCs^8,42^. Furthermore, the antioxidant activity of both CO and CH cannot be ignored^84,86^. CO and its main constituent (eugenol) play a main role in the modulation of redox status by decreasing the formation of toxic metabolite acrolein and increasing the level of anti-oxidant enzymes together with its free radical-scavenging activity^87–89^. CH-NPs demonstrate redox activity owing to increasing intracellular antioxidant enzymes in biological systems (GSH and SOD), prevention of ROS production, and inhibition of lipid oxidation^90^. Considerably, the biomarker assessment was in agreement with the histopathological examination of dissimilar rat groups, substantiating the improved urothelial protective effectiveness of the optimal formula (F2) against CP urotoxicity.

Collectively, the results of FT-IR and DSC confirmed the drug entrapment in the NSLCs. TEM showed the nanoparticles as a scaffold for the drug with the external CH layer. In vivo studies on experimental animals proved the prophylactic uro-protective effect of the optimal formula (F2). The remarkable uroprotective effect of the optimal formula (F2) against CP-induced HC in the rat model was verified by the aforementioned in vivo results, which were all compatible with one another. Several related causes, including the following, may be responsible for such apparent activity: (1) APO and CO both have anti-oxidant and anti-inflammatory effects, demonstrating their pharmacological activity on HC^9,87^; (2) the mucoadhesive property of CH can encompass the residence period of the NSLCs, hence extending APO and CO release^55^; (3) CH’s permeability boosting characteristics might enhance NSLCs transcellular and paracellular intake by reversible opening the tight junctions across and between the epithelial cells^91,92^. From all of the aforementioned results, it seems that the inclusion of APO into CO-based NSLCs wrapped in CH is considered as an alternative prophylactic therapy for patients suffers from HC triggered by CP administration. Moreover, the promising results achieved during studying cytotoxicity on the T24 cell line, encourage their subsequent application as an adjuvant in protocols of bladder cancer treatment. These results motivate the use of phytopharmaceuticals for the treatment of different diseases by their prospective formulation in the form of actual medicines.

## Conclusion

For the first time, a promising combination of two golden phytopharmaceuticals (APO and CO) as NSLC systems with tunable PS, narrow PDI, and high EE% was accomplished using an emulsification ultrasonication technique. APO-loaded CO-based NSLCs demonstrated high stability, sustained release, and cytotoxicity against the T24 bladder cancer cell line. Moreover, oral pretreatment with the optimal formula (F2) conferred an enormous in vivo uroprotective effect contra CP-induced HC in rats. The results obtained in the contemporary study were motivating enough to highlight the feasibility of using natural phytopharmaceutical in therapy.

### Supplementary Information

Supplementary Information.

## Data Availability

The datasets generated during and/or analyzed during the current study are available from the corresponding author on reasonable request.
