# Supplementary material for: Quality by design approach of apocynin loaded clove oil based nanostructured lipid carrier as a prophylactic regimen in hemorrhagic cystitis in vitro and in vivo comprehensive study
Source: Sci Rep. 2024 Aug 19;14:19162. doi: 10.1038/s41598-024-68721-z (PMC11333711; doi:10.1038/s41598-024-68721-z)
Supplement: Supplementary file 1 — Supplementary Information. [file 41598_2024_68721_MOESM1_ESM.docx]

**Supplementary table**

| **pH** | **Formula** | **Zero- order** | | **First order** | | **Higuchi diffusion** | | **Korsmeyer-Peppas** | | | **Main transport mechanism** | **Weibull** | | |
| --- | --- | --- | --- | --- | --- | --- | --- | --- | --- | --- | --- | --- | --- | --- |
|  |  | **R^2^** | **AIC** | **R^2^** | **AIC** | **R^2^** | **AIC** | **R^2^** | **AIC** | **n** |  | **R^2^** | **AIC** | **β** |
| **1.2** | **APO aqueous solution** | 0.767 | 39.01 | 0.910 | 29.90 | 0.953 | 28.97 | 0.884 | 4.45 | 0.294 | Fickian | 0.927 | -41.60 | 0.416 |
| **6.8** |  | 0.838 | 67.93 | 0.900 | 55.93 | 0.947 | 51.37 | 0.938 | 53.36 | 0.783 | Non Fickian | 0.946 | -29.55 | 0.851 |
| **7.4** |  | 0.646 | 82.27 | 0.805 | 59.52 | 0.834 | 55.00 | 0.803 | 5.78 | 0.993 | Non Fickian | 0.872 | -16.00 | 0.644 |
| **1.2** | **Optimal formula** **(F2)** | 0.893 | 12.38 | 0.890 | 11.87 | 0.837 | 17.22 | - | - | - | - | - | - | - |
| **6.8** |  | 0.945 | 34.10 | 0.950 | 30.55 | 0.940 | 34.85 | - | - | - | - | - | - | - |
| **7.4** |  | 0.911 | 61.63 | 0.954 | 52.49 | 0.970 | 43.04 | 0.926 | 43.78 | 0.580 | Non Fickian | 0.952 | -39.11 | 0.714 |

**Table S1**: Kinetic analysis of the drug release data of APO aqueous solution as well as the optimal formula (F2), (See **Table 2** for **F2** composition)

**(R^2^)**: Coefficients of determination.

**(AIC)**: Akaike information criterion.

**(n):** Diffusional exponent.

**(β):** Shape parameter.

**Supplementary figures**


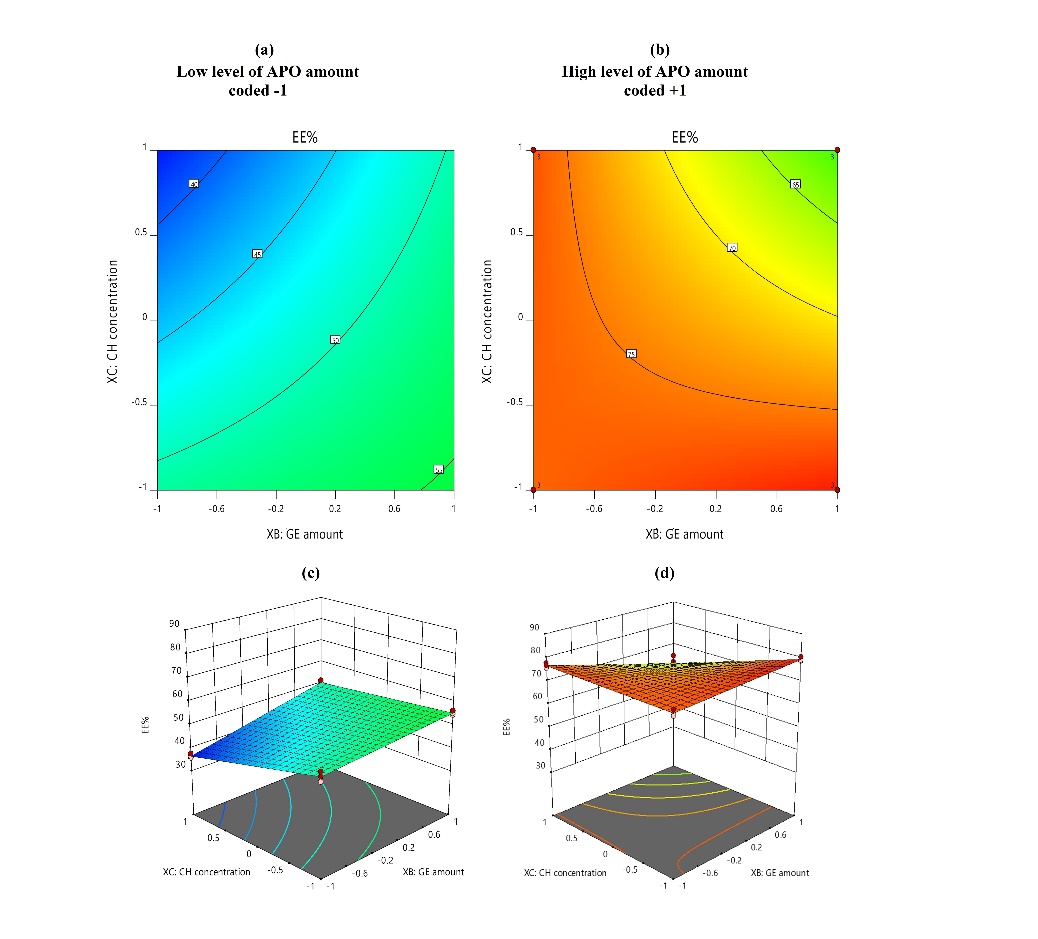


**Fig. S1**. Contour (A-B) and 3-D (C-D) plots representing the effect of the interaction between the amount of GE (B) and concentrations of CH (C) on EE (%), at the low and high levels of APO (A), respectively


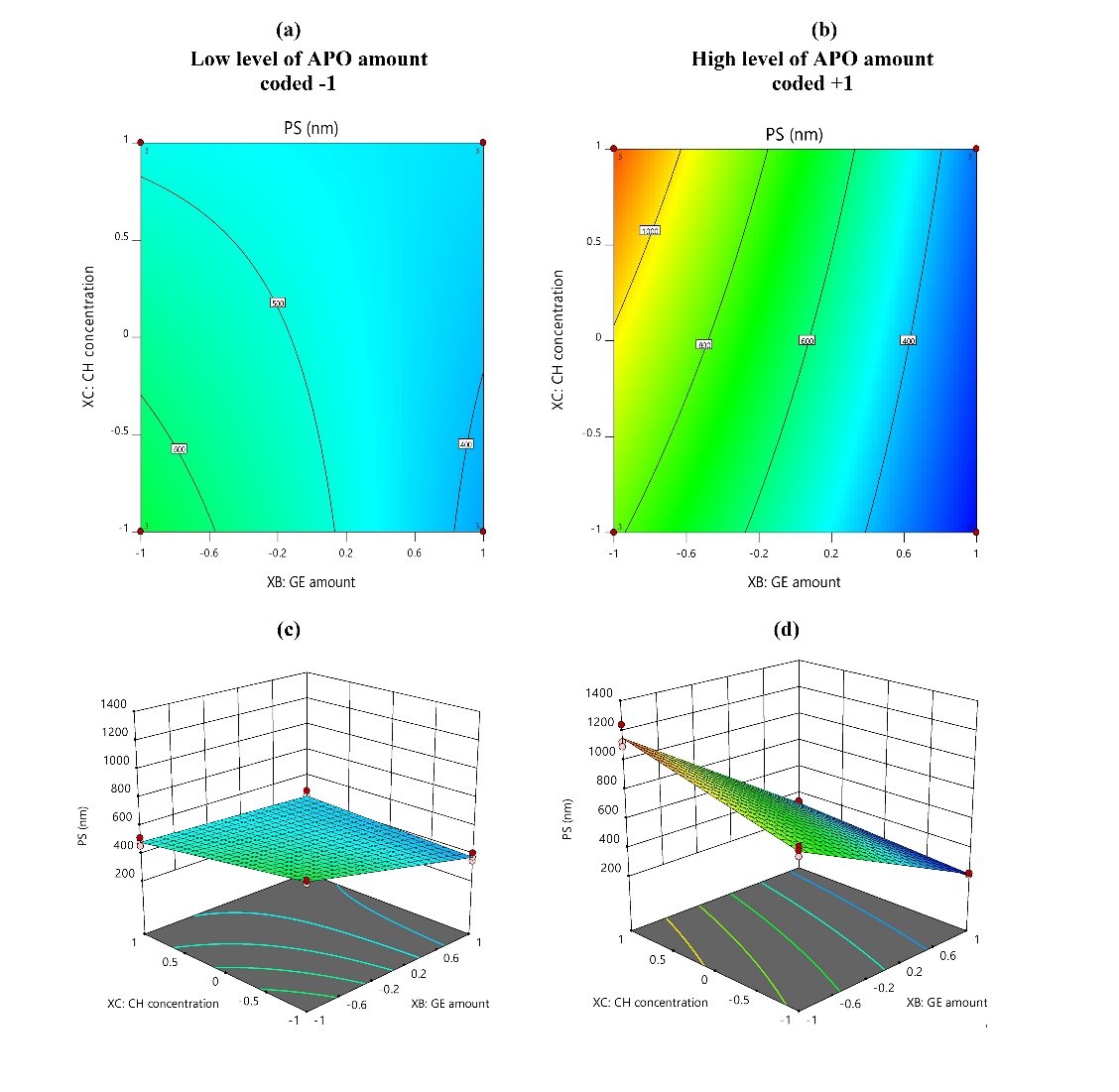


**Fig. S2**. Contour (A-B) and 3-D (C-D) plots representing the effect of the interaction between the amount of GE (XB) and concentrations of CH (XC) on PS (nm), at the low and high levels of APO (XA), respectively


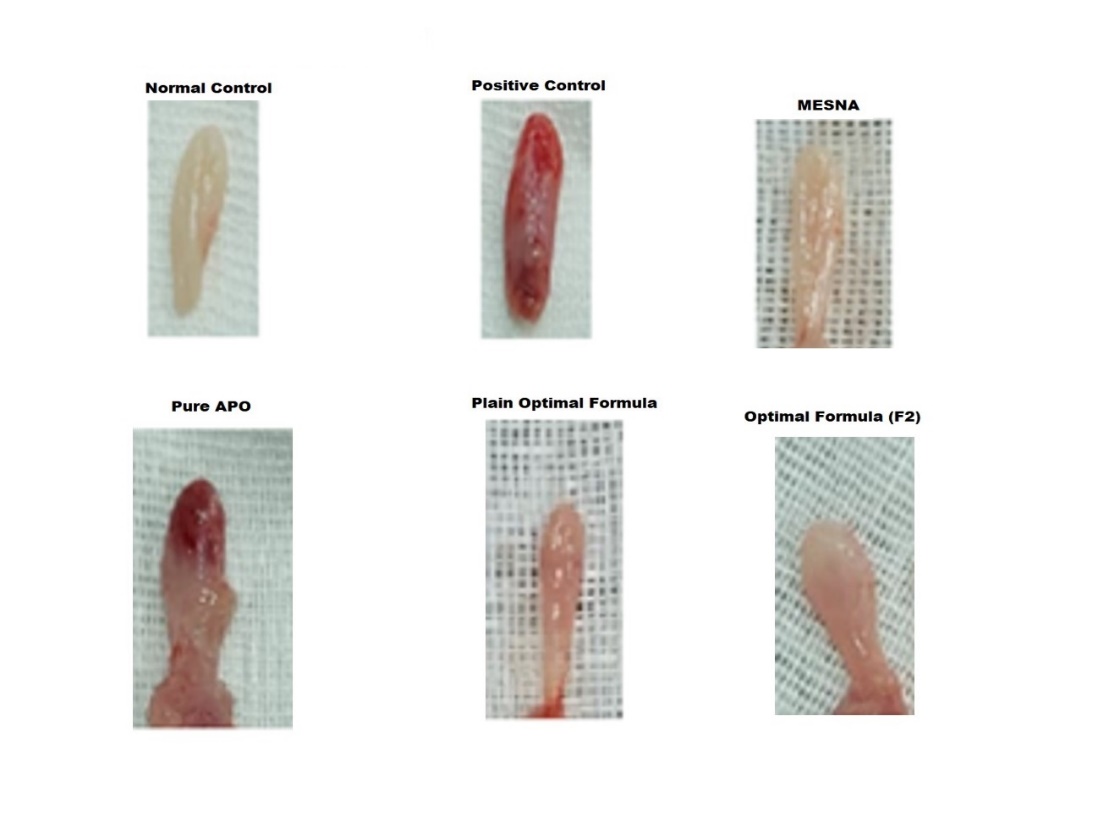


**Fig. S3**. Macroscopic changes for rat's bladders subjected to pretreatment regimen with different investigated formulations against CP-induced HC experimental model.


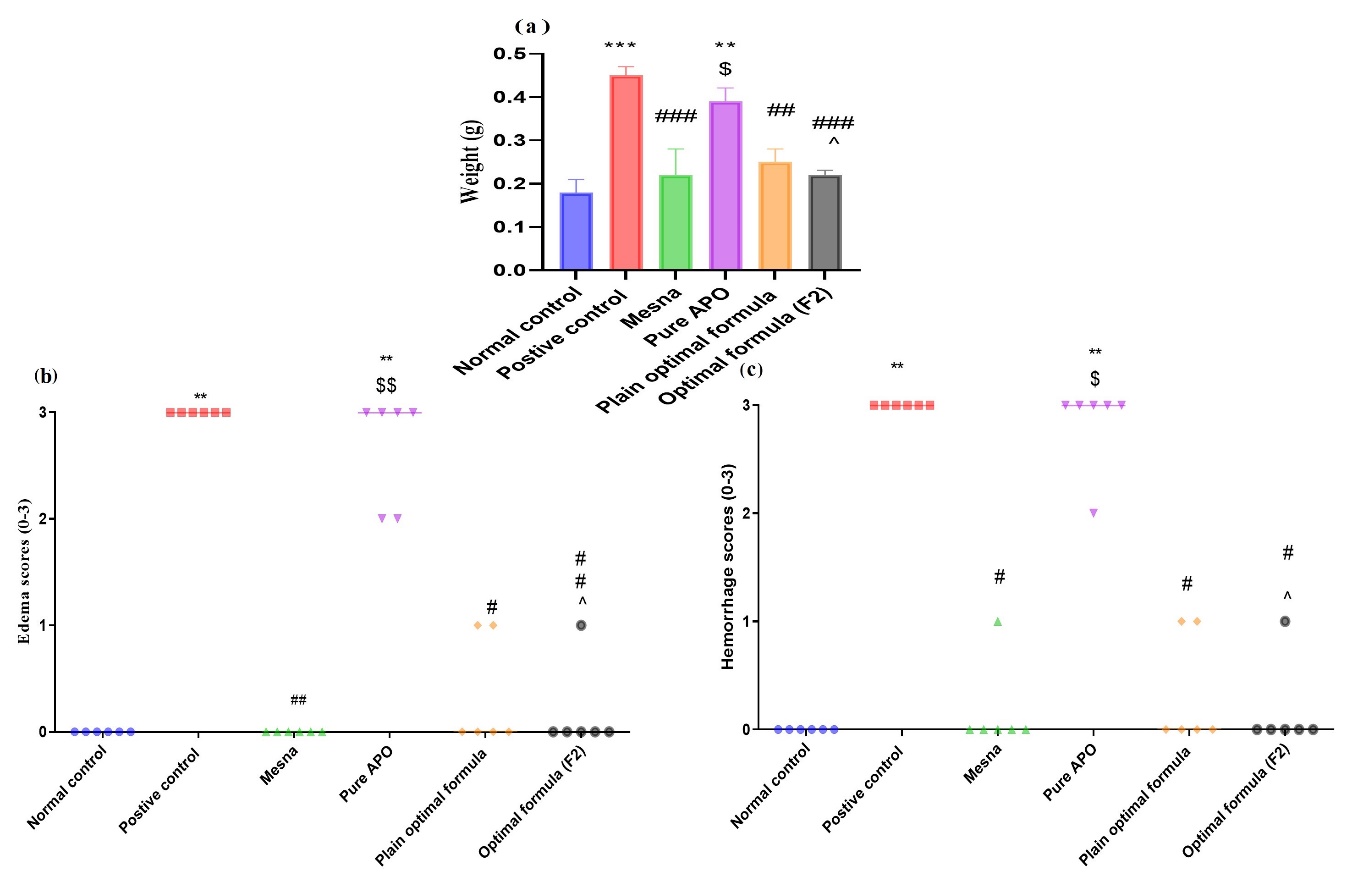


**Fig. S4**. Macroscopic evaluation parameters (A) weight, (B) edema and (C) hemorrhage for rat's bladder subjected to pretreatment regimen with different investigated formulations against CP-induced HC experimental model, (n=6).

Statistical significances of rat groups are indicated as: ^**^*p< 0.01* and ^***^*p< 0.001* vs normal control, ^#^*p< 0.05*, ^##^*p< 0.01* and ^###^*p< 0.001* vs positive control, ^$^*p< 0.05* and ^$$^*p< 0.01* vs MESNA group and *^p < 0.05* vs pure APO group. One-way analysis of variance (ANOVA) parametric test was applied ensued by Tukey-Kramer multiple comparison test (**Fig.10 A**), while Kruskal-Wallis non parametric test was applied ensued by Dunn multiple comparison test **(Fig.10B-C).**
